# The Relationship between Nanostructured Bio-Inspired Material Surfaces and the Free Energy Barrier Using Coarse-Grained Molecular Dynamics

**DOI:** 10.3390/biomimetics8060453

**Published:** 2023-09-25

**Authors:** Fan Meng, Noriyoshi Arai

**Affiliations:** Department of Mechanical Engineering, Keio University, Yokohama 2238522, Japan; mengfan@keio.jp

**Keywords:** bio-inspired materials, water droplet, nanostructured solid surface, many body dissipative particle dynamics

## Abstract

Bio-inspired (biomimetic) materials, which are inspired by living organisms, offer exciting opportunities for the development of advanced functionalities. Among them, bio-inspired superhydrophobic surfaces have attracted considerable interest due to their potential applications in self-cleaning surfaces and reducing fluid resistance. Although the mechanism of superhydrophobicity is understood to be the free energy barrier between the Cassie and Wenzel states, the solid-surface technology to control the free energy barrier is still unclear. Therefore, previous studies have fabricated solid surfaces with desired properties through trial and error by measuring contact angles. In contrast, our study directly evaluates the free energy barrier using molecular simulations and attempts to relate it to solid-surface parameters. Through a series of simulations, we explore the behavior of water droplets on surfaces with varying values of surface pillar spacing and surface pillar height. The results show that the free energy barrier increases significantly as the pillar spacing decreases and/or as the pillar height increases. Our study goes beyond traditional approaches by exploring the relationship between free energy barriers, surface parameters, and hydrophobicity, providing a more direct and quantified method to evaluate surface hydrophobicity. This knowledge contributes significantly to material design by providing valuable insights into the relationship between surface parameters, free energy barriers, and hydrophilicity/hydrophobicity.

## 1. Introduction

Bio-inspired materials, which are inspired by living organisms, have emerged as a promising avenue for the development of advanced functions in various scientific disciplines [1,2,3]. Among these materials, bio-inspired superhydrophobic surfaces are gaining attention for their potential applications in self-cleaning surfaces and reducing fluid drag [4]. The compound eyes of mosquitoes [5,6], the tiny hairs of lotus leaves [7,8], and the leg structures of water striders [9] are typical examples of superhydrophobic structures found in nature that exhibit significant hydrophobic properties. In this context, scientists aim to explore the nanostructural intricacies of these bio-inspired materials to understand the underlying mechanisms responsible for their unique properties. The ability to mimic the hydrophobic properties of these natural structures opens up new possibilities for a wide range of practical applications, from anti-fouling coatings to efficient fluid transport systems [1,10].

A thorough understanding of the Wenzel [11] and Cassie [12] states is essential when studying surface hydrophobicity. These two states are crucial in describing the wetting behavior of droplets on rough or textured surfaces. In the Wenzel state, the droplet wets the surface completely, spreading over the entire roughness and making contact with the bottom of the surface structure. On the other hand, the Cassie state is characterized by the droplet resting on top of the surface structure, with only the tips of the surface features in contact, and there may be air pockets trapped between the droplet and the bottom of the surface structure. Although the Cassie state is highly hydrophobic, the Wenzel state has a lower equilibrium free energy than the Cassie state, and there is a large free energy barrier between the two states, so that a drop in the Wenzel state cannot spontaneously return to the Cassie state. This free energy barrier is therefore the dominant factor in the design of superhydrophobic materials.

Leroy and Müller-Plathe’s pioneering work proposed that surface hydrophilicity/ hydrophobicity could be tuned by controlling the length and depth of the roughness pattern [13]. Their research was instrumental in elucidating the relationship between surface parameters and the wetting behavior of materials. However, a limitation of their study was the lack of a quantified evaluation standard to accurately measure these effects. Colin and Parkin suggested that the design of superhydrophobic surfaces should focus on two main features: low surface energy to achieve a contact angle above 90${}^{\circ }$ on a flat surface and high surface roughness to increase the hydrophobicity of the surface [14]. For hydrophobic polymer films, hydrophobicity is evaluated using contact angle and surface energy [15]. The current study relies on contact angle and surface energy as the primary evaluation criteria. The contact angle serves as a common indicator to measure the contact state of liquid droplets on the surface of a material, providing insight into the interaction between the surface of the material and the liquid. If the surface of the material is hydrophobic, the droplets exhibit a high contact angle, indicating a preference for sliding across the surface rather than making direct contact. On the other hand, surface energy plays a pivotal role in characterizing the surface properties of a material, with low surface energy typically correlating with hydrophobic behavior [16]. As a result, these evaluation criteria have been widely used in research and application to assess the hydrophobicity of various materials. Quéré et al. introduced an analytical model to investigate the impact of surface parameters on hydrophobicity and contact angles [17]. Their work highlighted the significant correlation between surface hydrophobicity and surface free energy. However, they did not establish a direct link between surface free energy barriers, surface parameters, and surface hydrophobicity.

However, as mentioned above, while the free energy barrier is the essence of the phenomenon, it is also a quantity that is difficult to measure directly. In recent years, there have been numerous instances of employing molecular simulations to investigate droplet behavior on surfaces [18,19,20,21]. Molecular simulations offer a comprehensive view of the entire droplet–surface interaction process, allowing for the precise and quantitative adjustment of surface parameters. Consequently, this approach facilitates a more in-depth exploration of the intricate interplay between surface parameters and droplet behavior on the surface. While many researchers have traditionally focused on investigating the relationship between droplet contact angles and surface energy, our research takes a unique perspective. We delve into the connection between surface parameters, free energy barriers, and material properties. This departure from the conventional methodology is characterized by the utilization of the free energy barrier as a fundamental reference standard to evaluation surface hydrophobicity. Therefore, our study goes beyond traditional approaches by exploring the relationship between free energy barriers, surface parameters, and hydrophobicity, providing a more direct and quantified method to evaluate surface hydrophobicity. Through a series of simulations, we investigate the behavior of water droplets on surfaces with varying surface parameters. We establish a profound relationship between surface parameters, free energy barriers, and material properties. By bypassing the reliance on conventional contact angle measurements, our research opens new avenues for the accurate assessment of surface hydrophobicity and provides valuable insights for material design and engineering.

This innovative approach allows more direct determination of the free energy barrier on the surface parameter and provides a deeper understanding of how to optimize materials for desired hydrophilic/hydrophobic properties, thereby streamlining material design processes.

## 2. Model and Methods

### 2.1. Many-Body Dissipative Particle Dynamics

We employed the many-body dissipative particle dynamics (MDPD) method [22,23,24] to investigate the relationship between the nanostructured solid surface and the free energy barrier. In the classical dissipative particle dynamics (DPD) method [25,26,27], the interaction between particles involves only repulsive forces that reflect the average of the forces between several particles [28,29]. Therefore, the classical DPD method cannot reproduce sharp density differences, such as gas–liquid interfaces. To overcome this drawback, attraction terms have been introduced into the DPD conservation forces as follows:

(1) $$F_{ij}^{C}=a_{ij}\left(1-\frac{\left|r_{ij}\right|}{rc}\right)n_{ij}+b_{ij}\left({\bar{\rho }}_{i}+{\bar{\rho }}_{j}\right)\left(1-\frac{\left|r_{ij}\right|}{rd}\right)n_{ij}.$$

Here, r is the position vector, $r_{ij}=r_{j}-r_{i}$, $n_{ij}=\frac{r_{ij}}{\left|r_{ij}\right|}$, ${\bar{\rho }}_{i}$ represents the local density at the particle, and rc and $r_{d}$ are cutoff distances used to determine the effective range of the force. The first term represents an attractive interaction, while the second term accounts for the many-body effect, behaving as a repulsive interaction. Therefore, the values of $a_{ij}$ and $b_{ij}$ are chosen to be negative and positive, respectively. The local density is calculated as follows:

(2) $${\bar{\rho }}_{i}=\sum_{i\neq j}\frac{15}{2\pi r_{d}^{3}}{\left(1-\frac{\left|r_{ij}\right|}{rd}\right)}^{2}.$$

In the current report, we utilize reduced units for the cutoff radius $r_{c}$, the particle mass *m*, and the energy $k_{B}T$, where *T* denotes the temperature and $k_{B}$ represents the Boltzmann constant. Thus, $r_{c}=m=k_{B}T=1.0$, and the time unit is defined as $\tau =\frac{\sqrt{mr_{c}^{2}}}{k_{B}T}=1.0$.

### 2.2. Simulation Model and Conditions

**Figure 1:**
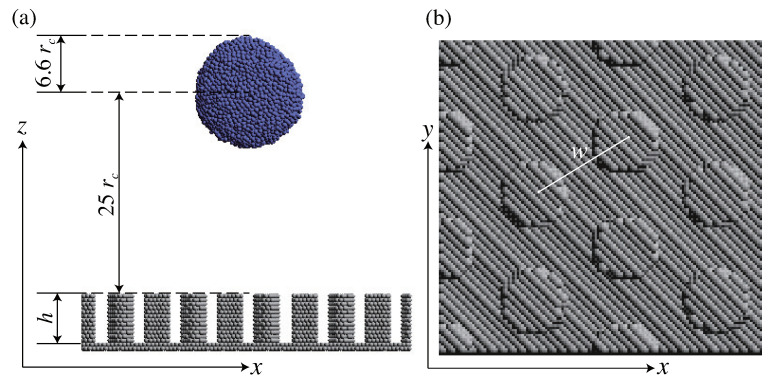
(**a**) Initial configurations of the droplet and the solid surface. The radius of the droplet, $r_{\mathrm{droplet}}$, is 6.6 $r_{c}$. The distance between the top of the pillar and the droplet is 25 $r_{c}$. The height of the pillar is defined as *h*. (**b**) Top view of the the mosquito eye model surface. The inter–center distance between the circles of each pillar is denoted as *w*.

In this study, the mosquito eye model, which is well-known for its superhydrophobic properties, was employed as a solid surface model for the nanostructured bio-inspired surfaces. We prepared ten wall models with different center-to-center distances (*w*) of the pillars and heights (*h*) of the pillars, as shown in Figure 1, to investigate the relationship between the Gibbs free energy barrier (ΔG) and the wall parameters. Table 1 presents a detailed overview of the particle numbers corresponding to each wall parameter.

Parameters $L_{x}$, $L_{y}$, and $L_{z}$ denote the dimensions of the simulation box along the *x*-, *y*-, and *z*-axes, respectively. They define the size of the computational domain in each direction and are critical for setting the boundaries within which the molecular dynamics simulations take place. Parameter *N* represents the total number of particles in the system. In the context of our simulations, it signifies the count of individual particles or molecules that interact with each other within the defined simulation box. These parameters are fundamental for configuring the simulation environment and understanding the scale of the molecular dynamics system. They ensure that the simulations are conducted within a well-defined space and with a specific number of interacting particles, allowing for accurate and reproducible results.

As depicted in Figure 1, a droplet is placed above the surface, and kinetic energy is imparted to the droplet in the downward direction to reproduce the solid surface–droplet collision process.

All simulations were performed in a constant volume and constant temperature ensemble. In the simulation, the thermostat is achieved using pairwise dissipative and random forces, which are coupled through the fluctuation–dissipation theorem. The parameters σ and γ represent dissipative and random forces, respectively, and satisfy $\sigma =\sqrt{2}\gamma k_{B}T$, reproducing a canonical ensemble. We set σ and γ to 3.0 and 4.5, respectively. The temperature was maintained at 0.5 $k_{B}T$ to effectively minimize the impact of thermal fluctuations.

Particles in the solvent are labeled as S, while particles in the wall are labeled as W. Based on our previous study [30], the interaction parameters in Equation (1) are defined as $a_{\mathrm{SS}}=-40k_{B}T$, $a_{\mathrm{SW}}=-25k_{B}T$, and $b_{\mathrm{SS}}=b_{\mathrm{SW}}=25k_{B}T$. We set the cutoff radius $r_{d}$ for the repulsive conservation force as 0.75 $r_{c}$ and the time step Δt as 0.005 τ. Each simulation run had a duration of 5000 τ to ensure the formation of a stable droplet.

## 3. Results

### 3.1. Measurement of the Free Energy Barrier

To ensure the accuracy of measuring the magnitude of the free energy barrier, a comprehensive series of simulations was conducted. The study involved varying the values of *w* and *h* each five times, with each value corresponding to five different $e_{k}$ values. In each $e_{k}$ value, over one hundred simulations were performed, resulting in a cumulative total of 2675 simulations. This extensive simulation setup aimed to explore the relationship between *w* and the free energy barrier ΔG.

In the context of our molecular dynamics simulations, $e_{k}$ represents the kinetic energy of the water droplet. Kinetic energy is a fundamental concept in physics and describes the energy associated with an object in motion. In our simulations, $e_{k}$ is a critical parameter as it governs the initial kinetic energy imparted to the water droplet before it impacts the surface under investigation. This kinetic energy influences the behavior of the water droplet upon impact, affecting its ability to transition between the Cassie and Wenzel states. An appropriate $e_{k}$ value is crucial, as it determines the conditions under which the droplet interacts with the surface features. Too much kinetic energy can lead to different outcomes than those observed under conditions with lower kinetic energy. Therefore, specifying $e_{k}$ accurately is essential to ensure the reliability and relevance of our simulation results. In order to accurately calculate the magnitude of the free energy barrier for each set of values of $w/r_{\mathrm{droplet}}$, for each set of values of $w/r_{\mathrm{droplet}}$, we give the droplet a certain initial kinetic energy and cause it to impact downward on the surface. It is a probabilistic event that the droplet will assume either the Wenzel state or the Cassie state after impacting the surface. According to Equation (4), in order to calculate the magnitude of the free energy barrier, we need to obtain the probability that the droplet transitions to the Wenzel state under the current parameters.

The droplet exhibits distinct free energies when in the Wenzel state and the Cassie state. Once trapped in the groove region, reaching the top of the pillars becomes more challenging due to the thermodynamically stable Wenzel state. Although the droplet possesses different free energies in the Wenzel state and the Cassie state, transitioning from the Cassie state to the Wenzel state involves crossing a barrier known as the free energy barrier. The magnitude of this barrier is closely linked to the surface parameters, and a higher barrier value typically indicates superior hydrophobicity of the material.

Throughout the simulations, the probability of Wenzel state formation was meticulously recorded for each $e_{k}$ value. By applying a computational formula (Koishi, 2009), the magnitude of the free energy barrier exhibited by the surface was accurately determined. The probability of the Wenzel state ($P_{w}$) is calculated using the following equation:

(3) $$P_{w}=P_{0}\exp\left(-\frac{\Delta G_{\mathrm{cw}}}{e_{k}}\right)$$

Here, $P_{0}$ represents the pre-exponential factor, $\Delta G_{\mathrm{cw}}$ is defined as the free energy barrier from the Cassie to Wenzel state, and $e_{k}$ corresponds to the kinetic energy of the center of mass of the droplet:

(4) $$e_{k}=\frac{1}{2}mv_{z}^{2}.$$

In our study, we conducted simulations to investigate the influence of two surface parameters, *w* and *h*, on the free energy barrier of the system. By systematically varying the values of *w* and *h*, we aimed to understand their impact on the free energy barrier.

**Figure 2:**
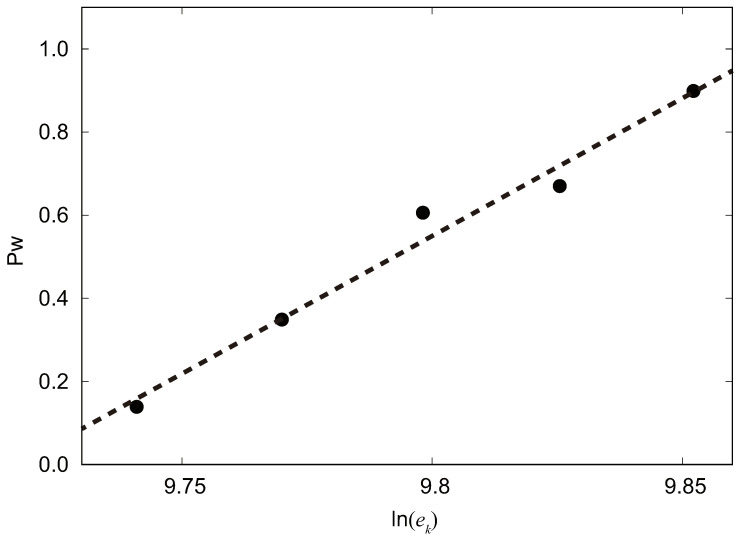
The *y*-axis represents the probability of the Wenzel state ($P_{w}$), while the *x*-axis corresponds to the natural logarithm of the kinetic energy ($e_{k}$).

We performed simulation experiments by varying the values of the solid surface parameter *w* at w=4.5, w=4.9, w=5.25, w=6.00, and w=6.60. In the case of w=5.25, we observed the probability of Wenzel state formation in the simulation results corresponding to the initial kinetic energy values of the droplet of 17,000, 17,500, 18,000, 18,500, and 19,000 to be 13.9%, 34.9%, 60.6%, 67.0%, and 90.0%, respectively, as shown in Figure 2.

**Figure 3:**
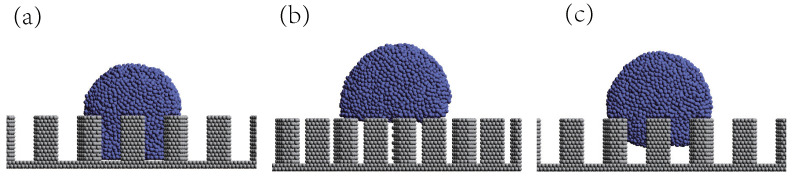
These are the three states of droplets on the surface: (**a**) Wenzel state, (**b**) Cassie state, and (**c**) middle Cassie state.

When either the *w* or *h* surface parameter surpasses a specific threshold, an intriguing phenomenon occurs, revealing the existence of an intermediate state situated between the Cassie and Wenzel states, as shown in Figure 3c. In this distinctive state, the droplet neither entirely occupies the upper surface of the pillars nor establishes direct contact with the surface base, deviating from the characteristics of the Wenzel state. Instead, a noticeable distance remains between the droplet and the surface bottom, which is smaller than the height *h* of the pillars. Interestingly, the static contact angle of the droplet in this intermediate state approaches that observed in the Cassie state. Therefore, in the context of our study, we classified this state as the Cassie state.

### 3.2. Effect on the Free Energy Barrier at Center-to-Center Distances (*w*) of the Pillars

**Figure 4:**
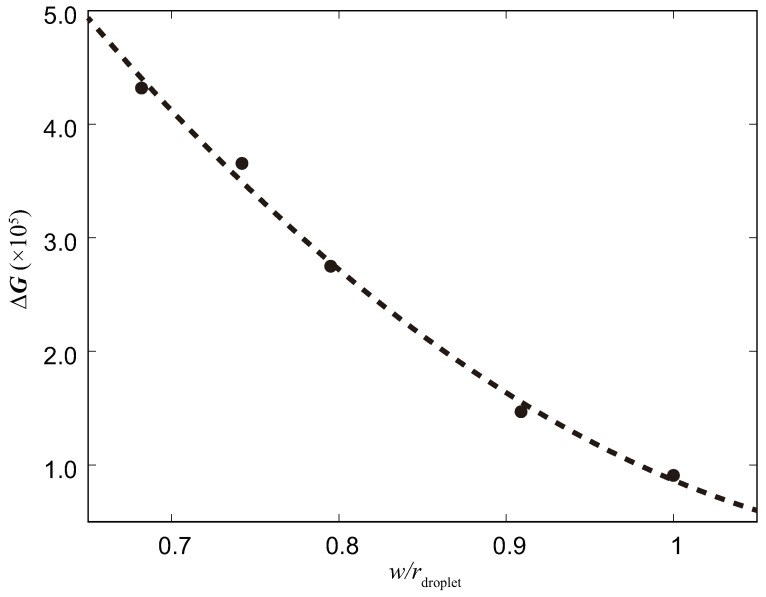
The effect of different *w* values on ΔG.

As the first series of simulations, we investigated the magnitude of the free energy barrier ΔG for different surface parameters *w*. We tried five different magnitudes of *w* based on the droplet radius: 0.68, 0.74, 0.80, 0.91, and 1.0 times. The free energy barrier curves determined using the computational approach outlined in Section 3.1 are shown in Figure 4. The dashed curve represents a quadratic curve fitted as $\Delta G=ax^{2}+bx+c$, where the constants of the fitting curves are a = $1.57143\times 10^{6}$, b = $-3.75542\times 10^{6}$, and c = $2.27064\times 10^{6}$.

The results clearly show that the magnitude of the free energy barrier exhibited by the surface increases rapidly as the distance between the surface columns decreases. Higher values of the free energy barrier imply higher hydrophobicity, indicating a significant relationship between surface parameters and the hydrophobic properties of the material.

### 3.3. Effect on the Free Energy Barrier at Height (*h*) of the Pillars

**Figure 5:**
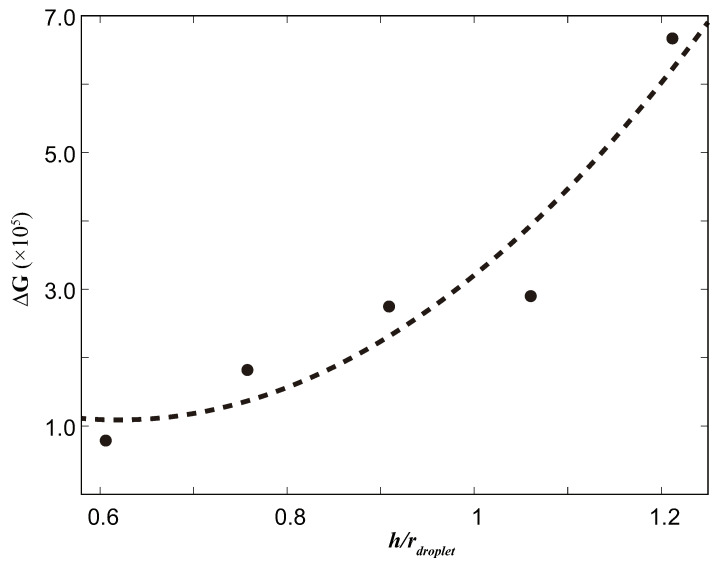
The effect of different *h* values on ΔG.

In the second set of simulations, we employed the same methodology to examine the magnitude of the free energy barrier for various surface parameters denoted as *h*. We specifically selected five distinct values of *h* = 4, 5, 6, 7, and 8. The computation-based approach outlined in Section 3.1 was employed to determine the free energy barrier curves, which are graphically represented in Figure 5. Furthermore, a dashed curve was fitted to the data using the quadratic equation $\Delta G=ax^{2}+bx+c$, where the fitted constants were found to be a = $1.57143\times 10^{6}$, b = $-3.75542\times 10^{6}$, and c = $2.27064\times 10^{6}$.

The results unequivocally demonstrate that as the height of the surface pillars increases, the magnitude of the free energy barrier on the surface experiences a significant rise. It is evident that altering the height of the surface pillars has a more pronounced impact on the free energy barriers compared to modifying the distance between them.

## 4. Discussion

From Figure 4, it is clear that the free energy barrier increases as the ratio of $w/r_{\mathrm{droplet}}$ decreases, indicating a smaller distance between the surface nanobumps. This suggests that a higher energy is required for the transition from the Cassie state (where the droplet is on top of the surface bumps) to the Wenzel state (where the droplet penetrates into the gaps between the bumps), making the transformation of droplets to the Wenzel state on the surface more difficult. In our study, we constructed the model based on the compound eyes of mosquitoes, which have very small distances between nanobumps. This finding is consistent with the research of Bhushan [7], as they observed that decreasing the distance between surface bumps increases the probability of air pocket formation and leads to an increase in the contact angle, indicating a more hydrophobic surface. These results support the simulation results in our study, where smaller values of *w* correspond to larger free energy barriers, indicating greater surface hydrophobicity.

The results obtained in this study show that ΔG increases quadratically as the space between the columnar structures decreases. It is important to note that our simulation model represents an idealized state; it models a perfectly uniform pattern in a mosquito eye structure with a narrow extra space. In real-world scenarios, the surface would have a more complex structure with varying column heights. In practical situations, reducing the spacing between the nanobumps would likely have a more pronounced effect on the free energy barrier of the surface than shown by the curve resulting from our simulations. Note that although these relationships held for ranges of w and h close to the droplet’s radius, it is not clear whether they hold for a narrower w or a higher h. This will be investigated in the future.

**Figure 6:**
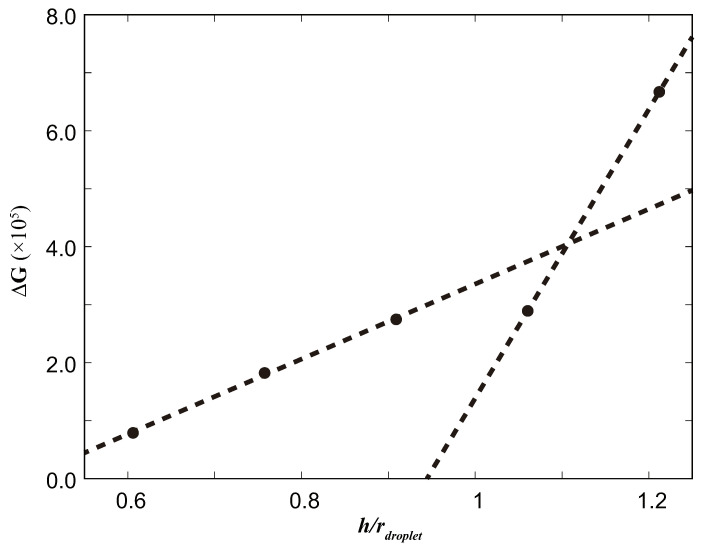
We fit two straight lines with the left three points and the right three points, respectively, to compare their slopes. We observe a significant change in the slope when the value of *h*/$r_{\mathrm{droplet}}$ approaches 1.

According to Extrand’s theory, the surface forces should exceed gravity (or other body forces) and act in an upward direction. In addition, the surface columns must be high enough to prevent the liquid from coming into contact with the bottom of the surface, which could cause the liquid to be pulled down and can lead to collapse. Figure 5 shows a significant increase in the free energy barrier as the height of the surface pillars (*h*) increases, particularly when the pillar height exceeds the radius of the droplet. This indicates that increasing the pillar height effectively increases the free energy barrier. Based on Figure 6, it is evident that the slope undergoes a significant change (approximately 3.85 times) before and after the surface pillar height becomes equal to the droplet radius. This observation may lead us to hypothesize that when the surface pillar height exceeds the liquid diameter, there is also a sudden increase in the free energy barrier. This is expected to be related to the position of the center of mass of the droplet and the height of the column. Specifically, the slope of Line 1 is $0.64\times 10^{6}$, while the slope of Line 2 is $2.49\times 10^{6}$. The slope is approximately 3.85 times higher. This indicates that the height of the surface pillar has a more pronounced effect on the surface free energy barrier. Furthermore, the effect of changing the height of the surface pillars appears to be more substantial than that of changing the distance between the pillars.

The results show that both *w* and *h* influence the magnitude of the free energy barrier. In the study by Burton and Bharat [31], it is observed that introducing a pattern on a flat polymer surface reduces adhesion and the coefficient of friction while increasing the contact angle, indicating a more hydrophobic surface. Similarly, Kwon et al. [32] demonstrate that the incorporation of hierarchical nanotextures on the surface increases its hydrophobicity. Both studies emphasize the positive effect of surface roughness and hierarchy on hydrophobicity.

**Figure 7:**
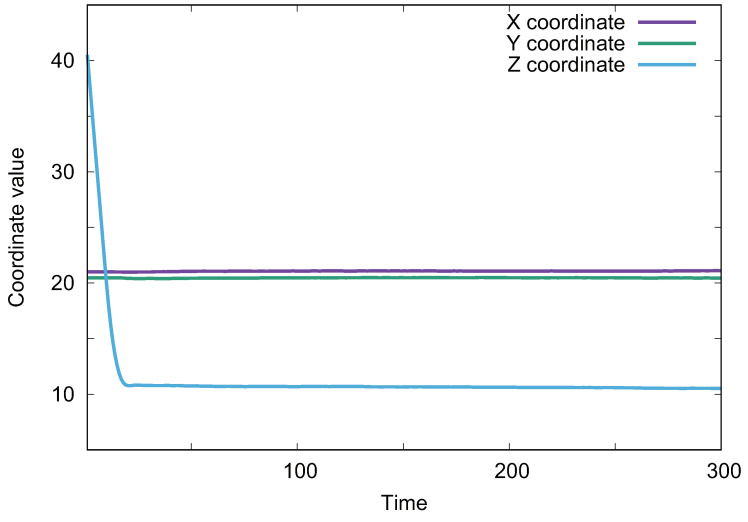
Droplet center coordinates vs. time.

An intermediate state, more typical in nature, becomes increasingly likely when we adjust the surface pillar height, *h*, to match the diameter of the droplet. We have calculated the relationship between the droplet center coordinates with time in Figure 7, and it can be seen that after a long period of time after the droplet impacts on the surface, the droplet center coordinates are almost unchanged; this can be basically recognized as a stable state. In this intermediate state, more than 70% of the droplet is usually fully immersed in the pillar structure, and sometimes even 100% of the droplet is fully immersed in the column structure, yet the droplet does not touch the lower end of the surface. This phenomenon aligns with David’s suggestion of a transitional state between the Cassie and Wenzel states [33]. We postulate that the frequently observed intermediate state in our superhydrophobic interface simulations represents this transitional phase.

**Figure 8:**
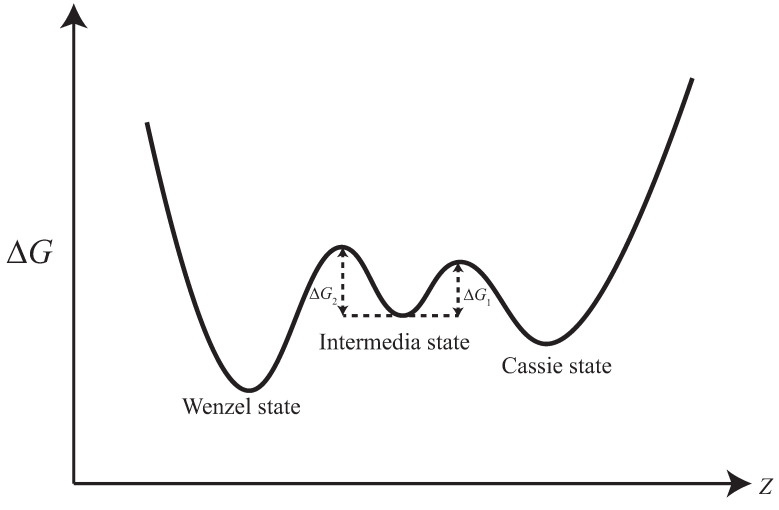
Our conjecture about the intermediate state free energy barrier.

Our conjecture regarding the energy landscape of this transition state is illustrated in Figure 8. In this situation, a droplet in the intermediate state has the potential to transition to either the Cassie or the Wenzel state. By providing the necessary energy stimulus to activate the droplet’s motion, it may be possible to manipulate the droplet in the intermediate state, making it more amenable to transitioning into either the Cassie or Wenzel state.

**Figure 9:**
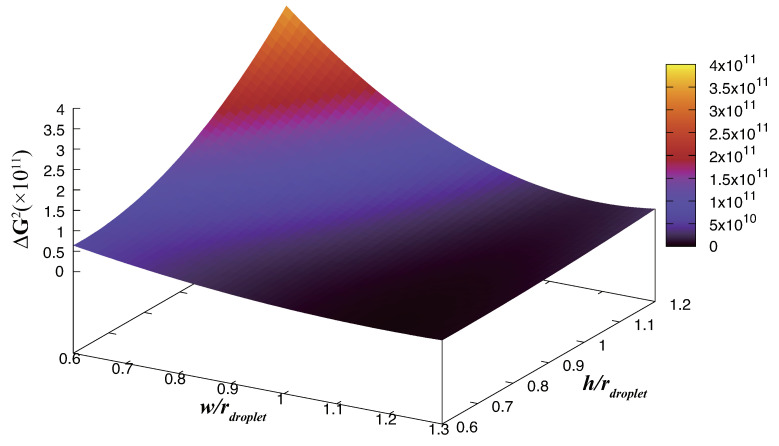
Integration of the *w*–*G* and *h*–*G* functions into a three-dimensional coordinate system.

We attempted to integrate the functions *w*–*G* and *h*–*G* into a three-dimensional coordinate system depending on the function (5), with the *x*- and *y*-axes representing the ratios of *w* and *h* to $r_{\mathrm{droplet}}$, and the *z*-axis representing $\Delta G^{2}$, as shown in Figure 9 Upon analysis, we observed that the highest point on the surface occurs when $w/r_{\mathrm{droplet}}$ is at its minimum and $h/r_{\mathrm{droplet}}$ is at its maximum. It is also evident that the rate of change of ΔG along the $h/r_{\mathrm{droplet}}$ axis is greater than the rate of change along the $w/r_{\mathrm{droplet}}$ axis. This observation suggests that designing the material to emphasize the height of the surface pillars is a more promising approach to increasing the hydrophobicity of the surface rather than focusing solely on adjusting the distance between the pillars. This approach not only adds depth to our research but also visualizes the intricate connection between surface parameters and the associated energy barriers, aiding with a comprehensive understanding of our study’s outcomes.

(5) $$\Delta G^{2}\left(w,h\right)=\Delta G\left(w\right)\;\cdot \;\Delta G\left(h\right)$$

Here, $\Delta G\left(w\right)=aw^{2}+bw+c$, and $\Delta G\left(h\right)=dh^{2}+eh+f$; a, b, c, d, e, and f are fitting parameters.

A unique aspect of our study is the ability to calculate the free energy barrier based solely on surface parameters. In previous research, surface experiments have typically been carried out to measure the contact angle of droplets on the surface, allowing an assessment of the hydrophilic or hydrophobic nature of the surface. In contrast, our study focuses on calculating the magnitude of the free energy barrier using only surface parameters, eliminating the need for experimental measurements. This provides significant convenience for material design, as it allows the direct determination of surface hydrophilicity or hydrophobicity based on the free energy barrier and without the need to perform experiments.

## 5. Conclusions

We performed coarse-grained molecular simulations to investigate the effects of droplets on solid surfaces with bio-inspired nanostructures with the aim of contributing to the advancement of solid-surface engineering. Our primary aim was to gain a deeper insight into the properties of these surfaces, focusing in particular on the nanostructure inspired by the compound eyes of mosquitoes, known as the mosquito eye structure. Through this investigation, we aimed to explore the influence of the physical properties of this nanostructure on the free energy barrier. Our results can be summarized as follows:

- The magnitude of the free energy barrier serves as an indicator of the hydrophilicity/hydrophobicity of the surface. In general, higher free energy barriers correspond to more hydrophobic surfaces, while lower free energy barriers correspond to more hydrophilic surfaces.
- When considering the physical properties alone without taking into account the surface chemistry, we observed that increasing the distance between the surface pillars results in a lower free energy barrier.
- Similarly, when considering only the physical properties and not the surface chemistry, we found that higher surface pillar heights lead to higher free energy barriers.
- The w−ΔG and h−ΔG curves obtained from our simulations provide predictive and evaluative capabilities for the free energy barrier of the surface. This allows us to predict and evaluate the hydrophilicity/hydrophobicity of the surface.

Our study provides valuable insights into the behavior of droplets hitting solid surfaces with bio-inspired nanostructures. By considering the physical properties of the nanostructure, we gain a better understanding of how these surfaces affect the free energy barrier. These findings have implications for the field of solid-surface engineering and offer potential applications in the design of surfaces with specific hydrophilic or hydrophobic properties.

## Figures and Tables

**Table 1:** Simulation conditions for each wall system.

*w*	*h*	$L_{x}$	$L_{y}$	$L_{z}$	*N*
450	6	36	35	60	67,728
490	6	39	38	60	71,460
525	6	42	40	60	74,640
600	6	48	46	60	82,656
660	6	52	51	60	90,132
525	4	42	40	60	66,512
525	5	42	40	60	74,416
525	7	42	40	60	90,224
525	8	42	40	60	90,448

## Data Availability

Not applicable.

## Abbreviations

The following abbreviations are used in this manuscript:

MDPD	Many-body dissipative particle dynamics
G	Gibbs free energy
